# Spread of clonal genovar E *Chlamydia trachomatis* among men who have sex with men

**DOI:** 10.1371/journal.pone.0259274

**Published:** 2021-10-29

**Authors:** Arabella Touati, Björn Herrmann, Nadège Hénin, Cécile Laurier-Nadalié, Cécile Bébéar, Bertille de Barbeyrac, Olivia Peuchant

**Affiliations:** 1 Department of Bacteriology, National Reference Centre for Bacterial Sexually Transmitted Infections, CHU Bordeaux, Bordeaux, France; 2 Section of Clinical Bacteriology, Department of Medical Sciences, Uppsala University, Uppsala, Sweden; 3 Infections Humaines à Mycoplasmes et Chlamydiae, EA 3671, Univ. Bordeaux, Bordeaux, France; University of Texas Health Science Center at San Antonio, UNITED STATES

## Abstract

In a previous study, we developed a Multi-Locus VNTRs Analysis (MLVA) typing system, called MLVA-5, for the discrimination of *Chlamydia trachomatis* genovar E strain. The results suggested the clonal spread of a MLVA-5 type 21 strain among men who have sex with men (MSM). We applied the MLVA-5 typing method on 157 French anorectal genovar E specimens and 19 Swedish specimens collected between 2010 and 2015. A total of 29 MLVA-5 types was obtained, with three predominant types among French samples: 78 specimens belonged to MLVA-5 type 21, two other types, 11 and 13, included 9 and 14 specimens, respectively. In 15 cases, one unique MLVA-5 type was observed for a single patient, 7 of which were new types not previously described. The distribution of MLVA-5 types according to sexual orientation showed that the 7 anorectal specimens from heterosexual patients belonged to 6 genotypes, and the 12 anorectal specimens from bisexual patients comprised eight types. The 95 anorectal specimens from MSM were distributed into 22 types, but 55 (57.9%) of them belonged to MLVA-5 type 21. Among the Swedish specimens from MSM, eight were from MLVA-type 21 (4 urines and 4 anorectal specimens). The results support the hypothesis of the spread of clonal genovar E strain among MSM.

## Introduction

Molecular epidemiological studies are essential for understanding the genetic population structure of *Chlamydia trachomatis*, to gain insight into the transmission of the bacterium among men who have sex with men (MSM) and to monitor emerging trends. In 2004, the French Institute for Public Health Surveillance launched sentinel surveillance for anorectal *C*. *trachomatis* infections released in 2010 by the French National Reference Center (NRC) for Chlamydia (Bordeaux, France) [1]. In this monitoring system, laboratories performed routine testing for *C*. *trachomatis*, and positive anorectal specimens were referred to the French NRC for specific detection of L or non-L strain [2]. In the latest case, genovar identification was performed by amplifying and sequencing the *omp*A gene directly from clinical specimens [3].

Data recorded in France by the NRC showed that genovars D, G, E, and J have constituted more than 90% of the non-L anorectal isolates between 2010 and 2014. Genovar E showed a constant increase since 2006 (de Barbeyrac et al. [Unpublished]) to stabilize around 23% since 2010.

Multi-locus molecular typing systems with higher discrimination than *omp*A sequencing have been developed, including multi-locus variable number tandem repeat (VNTR) analysis (MLVA). We previously developed this typing system, called MLVA-5, for *C*. *trachomatis* to enable the differentiation and subgrouping of isolates within a genovar [4]. This method provided high resolution for the differentiation of *C*. *trachomatis* strains of genovar E [4]. Interestingly, we showed that the majority (9/11) of anorectal *C*. *trachomatis* genovar E isolates collected between 2006 and 2010 displayed the same and unique MLVA-5 type 21, which was not the case for the *C*. *trachomatis* genovar E urogenital isolates tested in this study [4]. All the nine patients were MSM and were mainly from Paris, suggesting clonal spread of the strain, but only few isolates were analyzed. To investigate the hypothesis that the observed increase in genovar E is associated with the dissemination of MLVA-5 type 21, we typed anorectal genovar E specimens collected in France between 2010 and 2014 using the MLVA-5 typing method. We also genotyped Swedish genovar E specimens obtained from MSM between 2014 and 2015 to compare MLVA-5 types in another European country with a similar *omp*A genovar distribution as those collected among MSM in France [5].

## Materials and methods

Anorectal *C*. *trachomatis*-positive specimens were collected in France as part of national sentinel surveillance for anorectal *C*. *trachomatis* infections approved by the French Data Protection Authority (CNIL, no. 10.362) [1]. For each patient sexual behavior data were collected after written consent was obtained.

The Swedish specimens from Uppsala University Hospital were collected as part of routine diagnostics and were anonymized. Informed consent is not needed for the use of microbiological samples in the quality assurance of diagnostic methods according to the Biobanks in Medical Care Act (2002:297). The collection and use of anonymized samples were approved by the Regional Ethical Review Board of Uppsala, Sweden (Dnr 2007/312).

All the specimens were single-patient specimens. MLVA-5 genotyping was performed as described [4]. The obtained data were analyzed using Peak Scanner software 2 (version 2.0; Applied Biosystems) to perform sizing and to calculate the number of repeats for each marker. An allele number string, based on the number of repeats at each locus, was assigned to all isolates. The calculated numbers of repeats were imported into BioNumerics software (version 7.6.2; Applied Maths) for further cluster analysis.

## Results

Overall, we collected 975 French non-L anorectal specimens between 2010 and 2014. Among them, 228 were of genovar E (range 20.4–24.3% according to the year). Full MLVA-5 types were obtained for 157 of these specimens, resulting in a success rate of 68.8% (157/228) (Table 1).

**Table 1:** French non-L anorectal specimens.

Number of specimens	2010	2011	2012	2013	2014	Total
**Total**	71	98	90	308	408	975
**Genovar E (%)**	16 (22.5)	20 (20.4)	21 (23.3)	75 (24.3)	96 (23.5)	228 (23.3)
**Full MLVA-5 types (%)**	13	10	15	58	61	157 (68.8)

Among the 157 anorectal specimens, 8 were from women (6 heterosexual, 1 bisexual, 1 unknown), 147 from men and 2 from patients with unknown sex and gender. Sexual orientation, available for 107/147 men, showed that 95 were MSM, 11 were bisexual and 1 was heterosexual. Most patients were from the Paris area (52.3%, 82/157) and the remainders were from other French cities (47.7%, 75/157).

**Fig 1:**
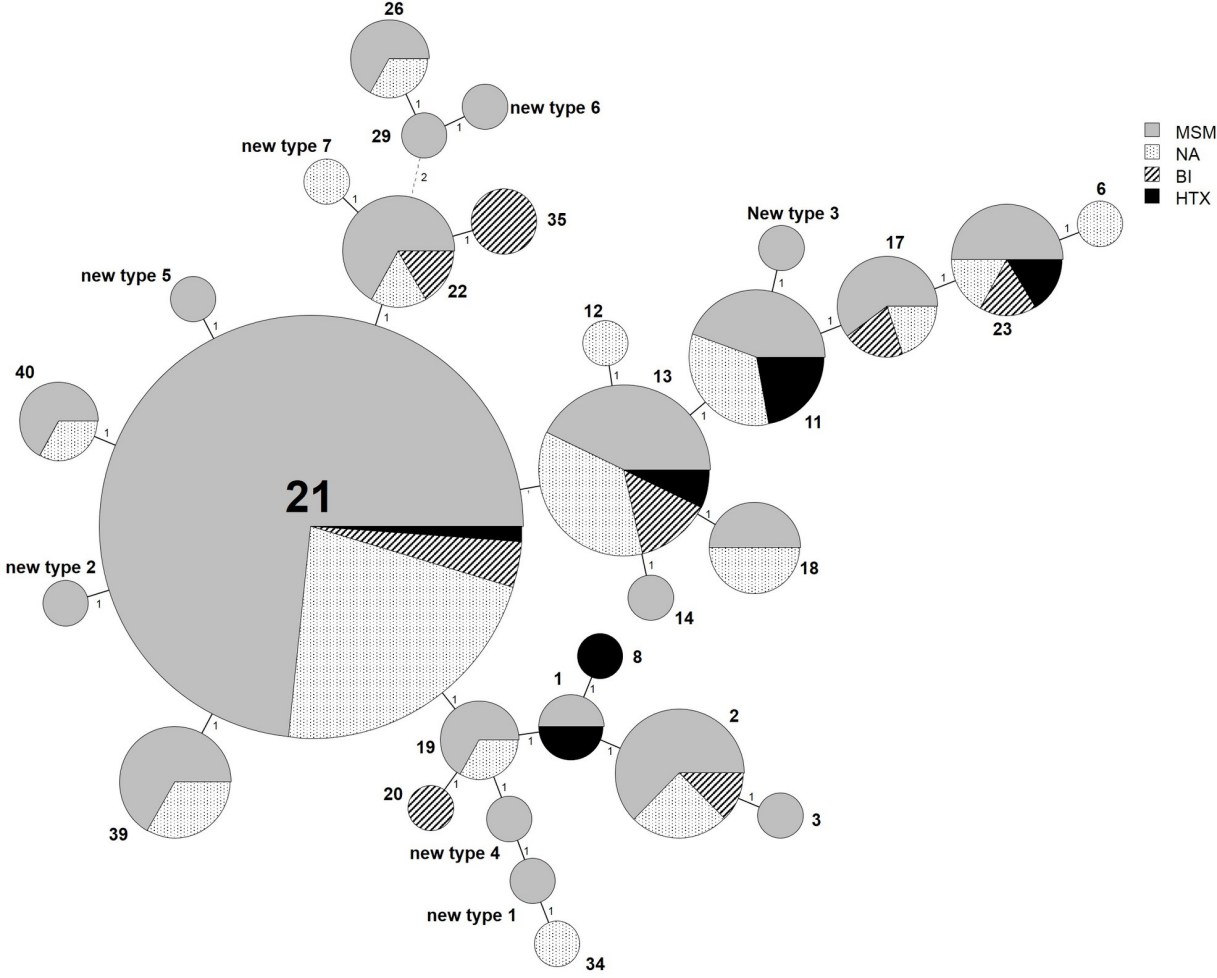
Minimum spanning tree of the MLVA-5 types of genovar E *C*. *trachomatis* clinical specimens according to sexual orientation. Each circle denotes a particular MLVA-5 type. The size of the circle is proportional to the number of specimens of the indicated MLVA-5 genotype. The distance between neighboring genotypes is expressed as the number of allelic changes. MSM: men who have sex with men (n = 110); BI: bisexual (n = 12), HTX: heterosexual (n = 7), NA: information not available (n = 43).

A total of 29 MLVA-5 types was obtained, with three predominant types: the MLVA-5 type 21 contained 78 specimens, types 11 and 13 included 9 and 14 specimens, respectively. In 15 cases, one unique MLVA-5 type was observed for a single patient, 7 of which were new types not previously described (Fig 1, S1 Table).

The distribution of MLVA-5 types according to sexual orientation showed that the 7 anorectal specimens from heterosexual patients belonged to six types (1, 8, 11, 13, 21 and 23), and the 12 anorectal specimens from bisexual patients comprised eight types (2, 13, 17, 20, 21, 22, 23 and 35). The 95 anorectal specimens from MSM were distributed into 22 types, but 55 (57.9%) of them belonged to MLVA-5 type 21, 6 (6.3%) to type 13, and 4 (4.2%) to type 11. The remaining 30 specimens were distributed into 19 types (for details see S1 Table). Of the 55 anorectal MSM cases with type 21, half were from the Paris area (49%, 27/55) whereas the remaining cases were from eight other French cities (51%, 28/55). These 55 anorectal specimens were collected over an extended period ranging between August 2010 and October 2014 with a variable distribution according to the years (2010 n = 6; 2011 n = 0; 2012 n = 7; 2013 n = 28 and 2014 n = 14). Nearly half of the specimens belonging to MLVA types 11 and 13 were from MSM (10/23, 43%), with a majority from Paris area (14/23, 60.8%).

Interestingly, the same distribution mentioned above for MSM, was also found for the 40 men with non-documented sexual orientation: 19/40 (47.5%) belonged to the MLVA-5 type 21, with a majority from the Paris area (63%, 12/19), 5 (12.5%) belonged to type 13, and 3 (4.2%) to type 11.

A total of 19 Swedish specimens from different patients, all MSM, were analyzed. A complete MLVA-5 profile was obtained for 15 specimens (two throat swabs, five urine samples and eight anorectal specimens). Four specimens were of type 2, one belonged to type 17, two to type 22 and eight (4 anorectal and 4 urines) were identified as MLVA-5 type 21.

The genetic relationships showed no obvious link between MLVA-5 type and year of specimen collection, patient age, geographic distribution, or HIV status.

## Discussion

The distribution of *omp*A genotypes among MSM differed overall from that previously described. We found that the predominant *omp*A genotypes were D, G, E and J, whereas studies elsewhere have shown the predominant MSM genovars to be G, D and J [6, 7]. The French *omp*A distribution bore a close resemblance to that among MSM in the UK [8]. Our results show a wide diversity of *C*. *trachomatis* genotype E strains as defined by MLVA-5 typing. However, nearly half of the isolates belonged to the MLVA-5 type 21, confirming that this type predominated among MSM in both France and Sweden. Two hypotheses may explain the predominance of one type: tissue tropism or epidemiological transmission patterns. Our results do not support the first hypothesis, because MLVA-5 type 21 was found in both urethral and anorectal Swedish specimens.

A comparative genomics realized by the team of Jeffrey *et al*. between the anorectal *C*. *trachomatis* E/150 strain which is of MLVA-5 type 21 (*in silico* genotyping) and the *C*. *trachomatis* E/11023 strain collected from cervical specimen revealed only 1130 substitutions and 54 insertions or deletions and the authors did not find any ORF correlated with rectal tropism in genovar E isolates [9]. Moreover, data based on multi-locus sequence typing of *C*. *trachomatis* strains give no support for tissue tropism, but indicate that sexual network structures explain the distribution of *C*. *trachomatis* strains in different anatomical locations in MSM [10, 11].

In conclusion, our results support the hypothesis of the clonal spread of MLVA-5 type 21 in the French and Swedish MSM population.

## Supporting information

S1 Table Distribution of the number of specimens according to MLVA-5 type. (XLSX)
